# Ionized calcium measurements during regional citrate anticoagulation in CRRT: we need better blood gas analyzers

**DOI:** 10.1186/s13054-015-1143-y

**Published:** 2015-12-09

**Authors:** Detlef Kindgen-Milles, Marlies Ostermann, Torsten Slowinski

**Affiliations:** Department of Anesthesiology, University Hospital Düsseldorf, Heinrich-Heine-University, Moorenstr. 5, D-40225 Düsseldorf, Germany; Department of Critical Care, King’s College London, Guy’s and St. Thomas’ Foundation Hospital, Westminster Bridge Road, London, SE1 7EH UK; Department of Nephrology, Charité Universitätsmedizin Berlin, Campus Mitte (CCM), Charitéplatz 1, D-10117 Berlin, Germany

Schwarzer et al. [1] showed discrepant postfilter ionized calcium concentrations [iCa] when using different blood gas analyzers (BGAs) and called for a change of the Fresenius regional citrate anticoagulation (RCA) protocol to ensure patient safety. Of note, precision of the BGAs was not tested.

In our opinion, the key message of their study is that commercially available BGAs are not accurate when measuring [iCa] outside the reference range and therefore clinicians should avoid using multiple BGAs to guide RCA in individual patients. However, there is no indication to change a RCA protocol which has been proven safe and effective in >10 studies including >2000 patients from different countries regardless of the BGA used [2–5].

Schwarzer et al. also raise concern about the potential risk of life-threatening citrate intoxication. Whether raised citrate levels are toxic or merely indicative of impaired cellular metabolism remains unclear, but excess citrate can cause metabolic alkalosis. The Fresenius RCA protocol and the technical specifications of the multifiltrate machine both include safety mechanisms to detect potential citrate accumulation early. The risk of citrate toxicity is low (<3 %), even in high-risk patients with liver failure [3, 5].

Given the proven advantages of RCA and the Kidney Disease Improving Global Outcomes (KDIGO) recommendation to use citrate as the first-line anticoagulant during continuous renal replacement therapy, the accuracy of commercially available BGA devices should be improved. In our opinion, there is no need to change a safe and effective protocol.

## Abbreviations

[iCa]: Ionized calcium concentration
BGA: Blood gas analyzer
KDIGO: Kidney Disease Improving Global Outcomes
RCA: Regional citrate anticoagulation
